# Whole exome sequencing in a child with acute disseminated encephalomyelitis, optic neuritis, and periodic fever syndrome: a case report

**DOI:** 10.1186/s13256-019-2305-3

**Published:** 2019-12-14

**Authors:** Pablo A. Ledesma, Juan Carlos Guerra, Manuel Burbano, Patricio Procel, Luis Alberto Pedroza

**Affiliations:** 1 Massachusetts General Hospital, 55 Fruit St, Boston, MA 02114 USA; 2 Harvard Medical School, 25 Shattuck St, Boston, MA 02115 USA; 3 Universidad San Francisco de Quito, Escuela de Medicina, Diego de Robles, Cumbaya, 170901 Quito, Ecuador; 4 Hospital de los Valles, Av. Interoceanica km 12.5 y Av. Florencia, Quito, Ecuador; 5 Baylor College of Medicine, 1 Baylor Plaza, Houston, TX 77030 USA

**Keywords:** Case report, Whole exome sequencing, Acute disseminated encephalomyelitis, ADEM, ADEM-ON, Optic neuritis, *NLRP12*, Periodic fever syndrome, Familial cold autoinflammatory syndrome, FCAS2

## Abstract

**Background:**

Acute disseminated encephalomyelitis is generally preceded by an infection, and it is usually self-limiting and non-recurrent. However, when there are multiple attacks of acute disseminated encephalomyelitis followed by optic neuritis, it is defined as acute disseminated encephalomyelitis-optic neuritis. To the best of our knowledge, there are no previous reports of acute disseminated encephalomyelitis and optic neuritis preceded by autoinflammation, triggered by periodic fever syndrome.

**Case summary:**

We report on a case of acute disseminated encephalomyelitis with optic neuritis and periodic fever syndrome in a 12-year-old Ecuadorian Hispanic boy with several relapses over the past 10 years, always preceded by autoinflammatory manifestations and without evidence of infectious processes. Whole exome sequencing was performed, and although the results were not conclusive, we found variants in genes associated with both autoinflammatory (*NLRP12*) and neurological (*POLR3A*) phenotypes that could be related to the disease pathogenesis having a polygenic rather than monogenic trait.

**Conclusion:**

We propose that an autoinflammatory basis should be pursued in patients diagnosed as having acute disseminated encephalomyelitis and no record of infections. Also, we show that our patient had a good response after 1 year of treatment with low doses of intravenous immunoglobulin and colchicine.

## Introduction

Multiple sclerosis (MS) and acute disseminated encephalomyelitis (ADEM) are autoinflammatory demyelinating diseases of the central nervous system (CNS). One of the main differences between these two entities is the chronicity and progressive neurological disability in MS, while ADEM is self-limiting, non-recurrent, and rarely produces neurological disabilities [1]. However, when there are multiple ADEM attacks followed by optic neuritis (ON), it is defined as ADEM-ON [2]. It is widely known that the clinical presentation of ADEM is an inflammatory process, and it is preceded by vaccination or infections. However, some cases are secondary to a fever of unknown origin [3, 4], which raises the question if a periodic fever syndromes (PFS) could be the recurrent trigger of the CNS inflammatory process in ADEM-ON as has been previously reported in MS [5]. *NLRP12*-associated autoinflammatory disorder (NLRP12AD) is a type 2 familial cold autoinflammatory syndrome (FCAS2), and part of the PFS [6–9]. It is an immune-mediated disease characterized by the presence of recurrent fever (of unknown origin) and other autoinflammatory features such as mouth ulcers and headaches [10]. Here, we describe a patient diagnosed as having ADEM-ON who also presented with familial cold autoinflammatory syndrome (FCAS) for whom we used whole exome sequencing (WES) to dissect possible variants in a non-HLA set of genes that could explain the patient’s clinical features (immunological and neurological).

## Case presentation

**Fig. 1:**
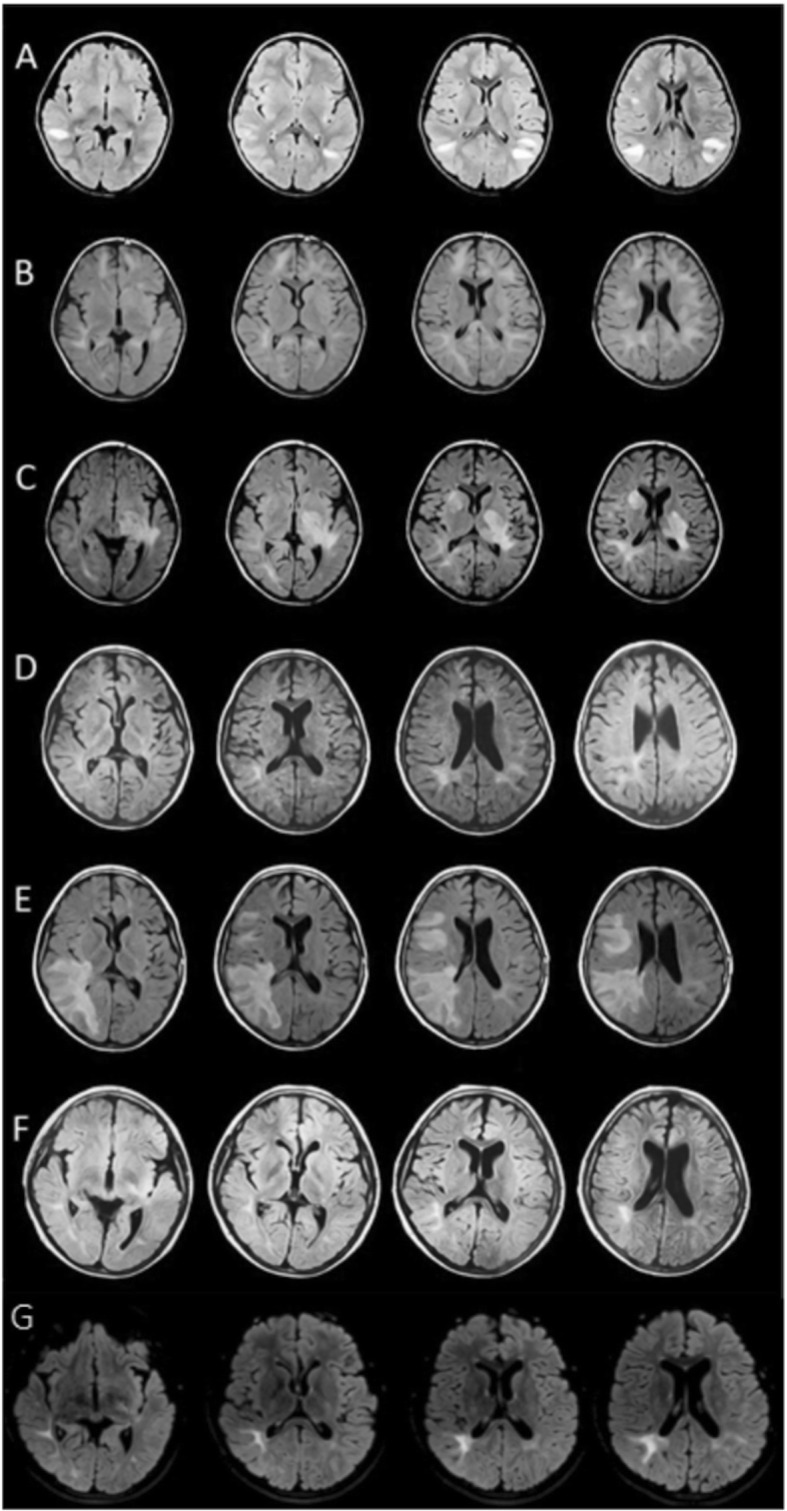
Fluid-attenuated inversion recovery T2 sequences showing multiple hyperintense lesions through the years. **a** 2008: Multiple injuries, mainly subcortical in temporal and occipitotemporal gyrus, and in the white matter of the corona radiata and semioval center. Lesions are enhanced after contrast. **b** 2010: Frontal subcortical injuries. Periatrial and parahippocampal lesions. There is a persistence of white matter lesions, and there are new infratentorial lesions. **c** 2011: Persistence of the periatrial lesions and left midbrain lesion. **d** 2013: Persistence of periatrial lesions and two small and new lesions in the basal ganglia infratentorial lesions. **e** 2014: New appearance of lesions in the cortex. Frontal and right superior temporal subcortical lesions. Right periatrial lesion has increased in size, reaching the cortex, parietal and occipital gyrus and corona radiata. **f** 2015: Persistence of periatrial lesions. There are smaller lesions in the white matter of the middle and lower right temporal gyrus and the semioval center. **g** 2017: After 7 months with intravenous immunoglobulin treatment – same lesions as the last magnetic resonance imaging in 2015. No new changes

Our patient is a 12-year-old Ecuadorian Hispanic boy from unrelated Hispanic parents; he presented to the pediatric department of the “Hospital de los Valles” with mouth ulcers, bilateral vision loss, headache, fever, lethargy, ataxia, dizziness, and left-sided hemiparesis. A clinical examination did not reveal any identifiable cause of fever. His familial history was unremarkable except for his maternal grandfather, who had type II diabetes mellitus. Our patient’s past medical history revealed a 10-year history of several episodes of pharyngitis, mouth ulcers, headaches, dizziness, fevers of unknown origin, and tonsillitis. These symptoms commonly preceded the appearance of neurological symptoms such as delayed speech, hypotonia, vision loss, ataxia, lethargy, and left hemiparesis. This pattern had been consistent and often required hospitalization for the treatment of neurological manifestations. The treatment consisted of corticoid therapy, which offered rapid improvement. Moreover, he has significant endocrine features, including small stature, delayed bone age, obesity, small hands, and hypogonadotropic hypogonadism. Although Prader–Willi syndrome was suspected, genetic analysis ruled this out. Ophthalmological imaging studies at his first hospitalization 10 years ago were consistent with a demyelinating and axonal lesion of the left optic tract, which is compatible with ON. In addition, C-reactive protein (CRP), an inflammatory index, was elevated at every hospitalization. The following laboratory studies, which were carried out on several occasions, had results within the normal range: complete blood count (CBC); serum chemistry; urine and blood culture; strep test; throat swab; serology for cytomegalovirus, (CMV), Epstein-Barr virus (EBV), herpes simplex viruses (HSV), rubella, and toxoplasmosis; immunologic screening for antinuclear antibodies, rheumatologic factor, immunoglobulin A (IgA) and immunoglobulin M (IgM) antiphospholipids; thyroid hormones; cortisol; and insulin. He also underwent the following examinations: chest and abdominal X-rays which were normal; pathergy test which was negative; brain magnetic resonance imaging (MRI) studies including fluid-attenuated inversion recovery (FLAIR) T2 sequences which showed multiple hyperintense lesions throughout the years (Fig. 1); spinal cord MRI studies that did not disclose any lesion; and cerebrospinal fluid analyses which were consistently normal with negative oligoclonal bands. MS was ruled out because he did not meet the McDonald diagnostic criteria for this disease. In addition, anti-myelin-associated glycoprotein (MAG), anti-aquaporin 4 (AQP4), and anti-myelin basic protein (MBP) were ordered but failed to disclose the diagnosis. Anti-myelin oligodendrocyte glycoprotein (MOG) was ordered, and the titer was 1:80 (considered negative); however, he was in treatment with intravenous immunoglobulin (IVIG) at the time of the anti-MOG evaluation. Given the unusual phenotype, we decided to perform a WES via a commercial service offered by the Baylor Miraca Genetics Laboratories. A list of variants potentially associated with the neurologic and immune features of our patient are listed in Table 1. A WES was not performed on his parents due to financial reasons. Even though the results were not conclusive, we found variants in genes associated with both autoinflammatory (*NLRP12*) and neurological (*POLR3A*) phenotypes that could be related to the disease pathogenesis being a polygenic rather than monogenic trait.

**Table 1:** Whole exome sequencing (WES) variants potentially associated with the neurologic and immunologic features of the patient

	Gene name	Isoform and nucleotide change (cDNA)	Amino acid change	dbSNP reference	SIFT/PolyPhen	Disease/Phenotype associated
Neurologic related diseases	*TRIM65*	NM_173547 c.1A>G	p.M1H	Novel variant	Damaging/Probably damaging	Probably associated to white matter hyperintensity PMID:25586835
	*POLR3A*	NM_007055 c.2934G>C	p.E978D	Novel variant	Damaging/Benign	Leukodystrophy, Hypomyelinating, 7, with or without oligodontia and/or hypogonadotropic hypogonadism (MIM:607694)
	*TSC2*	NM_000548 c.2445G>T	p.M815I	Novel variant	Tolerated/Possibly damaging	Tuberous sclerosis (MIM:613254)
	*GLI2*	NM_005270 c.67G>T	p.A23S	Novel variant	Tolerated/Benign	Holoprosencephaly 9 (MIM:610829); Culler-Jones syndrome (MIM:615849)
	*LAMA1*	NM_005559 c.7724C>T	T2575M	rs76482057	Damaging/Benign	Poretti-Boltshauser syndrome (MIM:615960)
	*LAMA1*	NM_005559 c.2808+5G>A	Splicing	rs201030108	NA**/**NA	Poretti-Boltshauser syndrome (MIM:615960)
	*NDUFA10*	NM_004544 c.1036G>A	p.E346K	Novel variant	Damaging/Benign	Leigh syndrome (MIM:256000)
Immunologic related diseases	*NLRP12*	NM_144687 c.910C>T	p.H304Y	rs141245482	Damaging/Probably damaging	Familial cold autoinflammatory syndrome (MIM:611762)
	*CSF3R*	NM_000760 c.2360A>G	p.Y787C	rs150281231	Tolerated/Benign	Neutrophilia, Hereditary (MIM:162830)
	*SIAE*	NM_170601 c.688C>T	p.R230W	rs200862001	Damaging/Probably damaging	Autoimmune disease (MIM:613551)
	*MCM4*	NM_005914 c.2063A>G	p.K688R	Novel variant	Tolerated/Benign	Natural killer cell and glucocorticoid deficiency with DNA repair defect (MIM:609981)

*cDNA* complementary deoxyribonucleic acid, *dbSNP* Single Nucleotide Polymorphism Database, *MIM* Mendelian Inheritance in Man, *NA* not available, *PolyPhen* Polymorphism Phenotyping, *SIFT* Sorting Intolerant From Tolerant

A final diagnosis of ADEM-ON and NLRP12AD was established. For the past year, our patient has remained on a monthly therapy with IVIG 500 mg/kg and orally administered colchicine (0.5 mg daily). With this treatment, he has remained free of new autoinflammatory and neurological episodes and has not required corticoids. An MRI study performed 7 months after the start of IVIG and colchicine showed an absence of new lesions.

## Discussion

ADEM is considered an autoinflammatory demyelinating disease of the CNS and is often secondary to infections [1]. However, some cases have been associated with recurrent inflammation and absence of known infections [3, 4], raising the question if autoinflammation could trigger CNS demyelination as has been previously reported in MS [5]. It could be expected to be of genetic origin – probably with a monogenic basis – based on the common origin of both diseases (that is, autoinflammation and ADEM-ON) and the early onset of manifestations. Although none of the variants can be considered to be the sole cause of the disease, we hypothesize that the presence of polymorphisms in *NLRP12* and *SIAE* (Table 1) trigger systemic autoinflammation, and such inflammation could influence the demyelination process in an unknown fashion. NLRP12AD, part of the cryopyrin-associated periodic syndromes (CAPS), has been associated with several autoinflammatory conditions that are similar to the immunological features of our patient [9–12]. However, to the best of our knowledge, there are no cases of NLRP12AD and inflammatory diseases in the CNS of humans. Interestingly, the role of *NLRP12* in inflammasome activation in the brain of murine models, including a model of experimental autoimmune encephalomyelitis, has been recently described [13–15]. In addition, *SIAE* has also been associated with susceptibility to autoimmune diseases [16]. However, this association has been questioned [17]. Although we failed to find a candidate gene or a genetic link with the neurological manifestations, the variant in *POLR3A* is important because bi-allelic mutations on this gene are associated with hypomyelinating leukodystrophy 7 (HLD7) [18]. Interestingly, our patient presents hypogonadotropic hypogonadism, which is one of the hallmarks of HLD7; however, the other clinical features and the MRI pattern are barely compatible with HLD7. Although only one of the *POLR3A* alleles is mutated, a new association between heterozygous mutations in *POLR3A* and susceptibility to varicella-zoster virus (VZV) infection (including encephalitis) was described recently. However, our patient did not show any evidence of VZV infection. Furthermore, these cases presented incomplete penetrance in healthy carriers [19]. Thus, we cannot rule out a possible influence of the *POLR3A* in the clinical features presented by our patient. High doses of IVIG are broadly used for the treatment of autoimmune diseases of different etiologies, including ADEM [20]. However, the use of low doses of IVIG in ADEM-ON has not been extensively documented. Recently, a cohort of patients with multiphasic disseminated encephalomyelitis (MDEM), of whom some received monthly IVIG treatment, was described [21]. These patients showed improved clinical manifestations, similar to our case. Our patient has had recurrent autoinflammatory symptoms leading to neurologic episodes every 6 months on average. Since the treatment with low-dose IVIG and colchicine was started, he has not presented any autoinflammatory or neurologic symptoms. It is known that IVIG at high doses works as an immunosuppressant to treat several autoimmune diseases. This effect is probably mediated by scavenging of complement and blockade or modulation of Fc receptors. At low doses, it is used as a prophylactic treatment in patients with immunodeficiencies in part by neutralizing the antigens. This could be a possibility in this patient because it could be neutralizing the virus or antigens. Therefore, this treatment prevents potential infections that usually trigger the autoinflammation and neurological manifestations, as is broadly known in ADEM or MDEM [1]. In any of these two scenarios, the IVIG and colchicine are preventing the inflammation that precedes the neurological manifestations. Typically, FCAS is treated with interleukin-1 (IL-1) inhibitors such as anakinra, rilonacept, or canakinumab [22, 23]. However, given the difficulty of finding these drugs in Ecuador and our patient’s economic inability to acquire them, colchicine was prescribed. This medication has a widespread effect on autoinflammatory disorders and has been widely accepted as a treatment in other PFS, such as in familial Mediterranean fever (FMF) [22]. Apart from some minor gastrointestinal side effects, the medication has been well tolerated by our patient, and he has not presented any autoinflammatory or neurological symptoms.

## Conclusions

To the best of our knowledge, this is the first case of *NLRP12*-associated autoinflammatory disease with neurological manifestations. We suggest that patients with ADEM-ON should be evaluated for autoinflammation (including variants in *NLRP12*) in the absence of documented infections.

## Data Availability

The datasets used and/or analyzed during the current study are available from the corresponding author on reasonable request.

## Abbreviations

ADEM: Acute disseminated encephalomyelitis
ADEM-ON: Multiple acute disseminated encephalomyelitis attacks followed by optic neuritis
AQP4: Aquaporin 4
CAPS: Cryopyrin-associated periodic syndromes
CBC: Complete blood count
CMV: Cytomegalovirus
CNS: Central nervous system
CRP: C-reactive protein
EBV: Epstein–Barr virus
FCAS: Familial cold autoinflammatory syndrome
FCAS2: Type 2 familial cold autoinflammatory syndrome
FLAIR: Fluid-attenuated inversion recovery
FMF: Familial Mediterranean fever
HLD7: Hypomyelinating leukodystrophy 7
HSV: Herpes simplex viruses
IgA: Immunoglobulin A
IgM: Immunoglobulin M
IL-1: Interleukin-1
IVIG: Intravenous immunoglobulin
MAG: Myelin-associated glycoprotein
MBP: Myelin basic protein
MDEM: Multiphasic disseminated encephalomyelitis
MOG: Myelin oligodendrocyte glycoprotein
MS: Multiple sclerosis
NLRP12AD: *NLRP12*-associated autoinflammatory disorder
ON: Optic neuritis
PFS: Periodic fever syndromes
VZV: Varicella-zoster virus
WES: Whole exome sequencing
